# An LPAR_**5**_-antagonist that reduces nociception and increases pruriception

**DOI:** 10.3389/fpain.2022.963174

**Published:** 2022-07-26

**Authors:** Jacqueline Langedijk, Erika Ivanna Araya, Amanda Ribeiro Barroso, Dagmar Tolenaars, Marc Nazaré, Hassane Belabed, Jens Schoene, Juliana Geremias Chichorro, Ronald Oude Elferink

**Affiliations:** ^1^Amsterdam University Medical Centers (UMC), Tytgat Institute for Liver and Intestinal Research, University of Amsterdam, Research Institute Amsterdam Gastroenterology, Endocrinology and Metabolism (AG&M), Amsterdam, Netherlands; ^2^Department of Pharmacology, Biological Sciences Sector, Federal University of Parana, Curitiba, Brazil; ^3^Departments of Chemical Biology and Structural Biology, Leibniz-Forschungsinstitut für Molekulare Pharmakologie, Berlin, Germany

**Keywords:** compound 3, hyperalgesia, inflammatory pain, itch (pruritus), LPAR5, nociception, TRPA1 (transient receptor potential A1)

## Abstract

**Introduction:**

The G-protein coupled receptor LPAR_5_ plays a prominent role in LPA-mediated pain and itch signaling. In this study we focus on the LPAR_5_-antagonist compound 3 (cpd3) and its ability to affect pain and itch signaling, both *in vitro* and *in vivo*.

**Methods:**

Nociceptive behavior in wild type mice was induced by formalin, carrageenan or prostaglandin E2 (PGE_2_) injection in the hind paw, and the effect of oral cpd3 administration was measured. Scratch activity was measured after oral administration of cpd3, in mice overexpressing phospholipase A2 (${\text{sPLA}}_{2}^{\text{tg}}$), in wild type mice (WT) and in TRPA1-deficient mice (*Trpa1 KO*). *In vitro* effects of cpd3 were assessed by measuring intracellular calcium release in HMC-1 and HEK-TRPA1 cells.

**Results:**

As expected, nociceptive behavior (induced by formalin, carrageenan or PGE_2_) was reduced after treatment with cpd3. Unexpectedly, cpd3 induced scratch activity in mice. *In vitro* addition of cpd3 to HEK-TRPA1 cells induced an intracellular calcium wave that could be inhibited by the TRPA1-antagonist A-967079. In *Trpa1 KO* mice, however, the increase in scratch activity after cpd3 administration was not reduced.

**Conclusions:**

Cpd3 has *in vivo* antinociceptive effects but induces scratch activity in mice, probably by activation of multiple pruriceptors, including TRPA1. These results urge screening of antinociceptive candidate drugs for activity with pruriceptors.

## Introduction

Lysophosphatidic acid (LPA) is a bioactive lipid involved in multiple functions like cell shape and migration, wound healing, platelet aggregation, lung fibrosis and hair growth, but it is also involved in pain and itch signaling (1–6). Intraplantar injection of LPA into the hind limb of mice showed dose-dependent nociceptive flexor responses (2). Intracellularly, LPA is the natural ligand of peroxisome proliferator-activated receptor gamma (PPARγ), whereas circulating LPA activates at least six different LPA receptors (LPAR_1−6_) (7–13). Neuropathic pain was shown to be mediated by LPAR_1_, as mice lacking *Lpar1* did not develop signs of neuropathic pain after peripheral nerve injury (4). However, recent studies have shown a prominent role of LPAR_5_ in neuropathic and inflammatory pain as well as in itch signaling (14–17).

Multiple LPAR_5_ specific antagonists have been developed to modulate the LPA/LPAR_5_ signaling pathway (17–21). In this study we focus on the LPAR_5_-antagonist compound 3 (cpd3), both *in vitro* and *in vivo*, in relation to sensory activity. Cpd3 (C_30_H_26_Cl_2_F_3_N_3_O_4_) is a non-lipid diphenylpyrazole derivative, which can prevent LPA-mediated activation in human mast cells (HMC-1) (IC50 0.141 μM) and mouse microglia cells (BV-2) (IC50 730 nM) (20). The compound is metabolically stable *in vivo*, with good absorption properties and is bioavailable after oral administration in mice.

Our research question was whether inhibition of LPAR_5_ by cpd3 would affect pain and itch perception in mice. Upon oral administration, cpd3 did have a reducing effect on inflammatory nociceptive behavior, induced by hindpaw formalin injection. In addition, mechanical hyperalgesia induced by carrageenan or PGE_2_ could be reduced with cpd3 administration. Strikingly however, we found that both in ${\text{sPLA}}_{2}^{\text{tg}}$ and WT mice, cpd3 induced a strong increase in scratch activity, which was dose-dependent. A possible explanation for this phenomenon was the activation of the itch-channel TRPA1 by cpd3, although administration of cpd3 to TRPA1-deficient mice still induced an increase in scratch activity upon cpd3 administration. These results suggest that an LPAR_5_-antagonist like cpd3 can have opposite effects on pain and itch perception, most likely by having opposite effects on noci- and pruriceptors.

## Materials and methods

### *In vitro* experiments

#### Chemicals

LPA 18:1 was purchased from Avanti; Cpd3 was a kind gift from the group of Marc Nazaré; DMSO from Merck, A-967079 from Sanbio; Glycofurol/tetraglycol and Solutol/Kolliphor from Sigma; PEG400 from Affymetrix; formalin from Alphatec; morphine from Merck S.A; carrageenan and PGE_2_ from Sigma.

#### Cell culture

HEK cells were cultured in Dulbecco's Modified Eagle Medium (DMEM; Lonza) containing phenol red (pH: 4,5g/L glucose), which was supplemented with L-glutamine (1%; Lonza). HMC-1 cells were cultured in suspension in DMEM/F12 Glutamax (Thermofisher). Both media were supplemented with penicillin/streptomycin (1%; Lonza), and fetal bovine serum (10% vol/vol; Gibco). Cells were cultured at 37°C, 10% CO_2_ in T75 and T160 culture flasks.

#### Calcium assay

**Figure 1:**
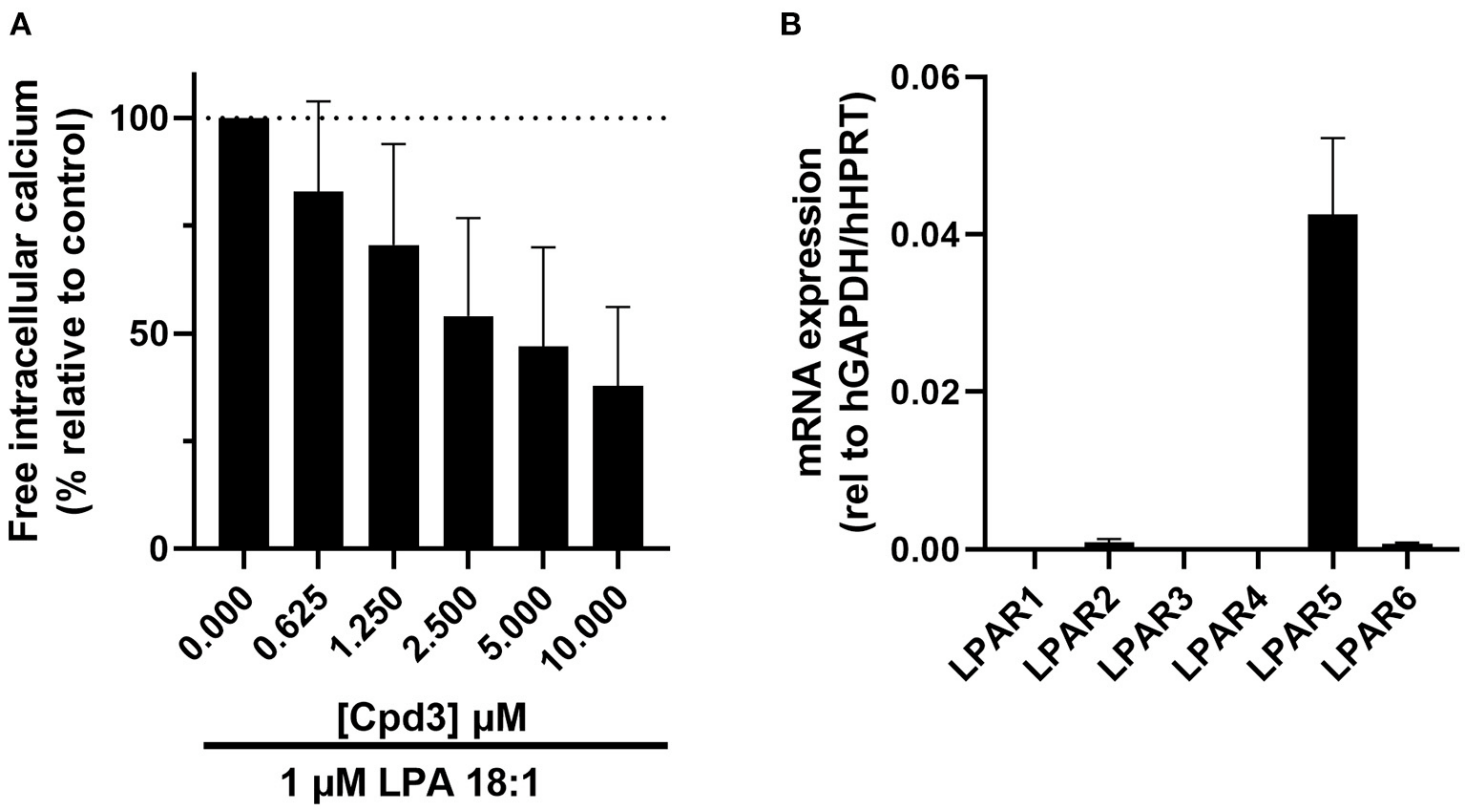
**(A)** Increase of free Ca^2+^ in HMC-1 cells, induced by 1μM LPA 18:1, was dose-dependently inhibited by cpd3 (maximal DMSO (vehicle) concentration 0.1%). Expressed in percentage of delta ratio relative to control **(B)** mRNA expression of LPA receptor 1-6 (*LPAR1-6*) in HMC-1 cells.

HMC-1 and HEK-TRPA1 cells were used for calcium measurements. Cells transduced with a doxycycline-inducible TRPA1 construct (22) were incubated with doxycycline (200ng/mL) 24 hours before start of the experiment. Attached cells were washed in HBSS without calcium, containing phenol red (Lonza) and trypsinized. They were resuspended in DMEM without serum and washed twice with HBSS containing calcium, without phenol red, buffered with 10mm HEPES. Cells were counted and diluted to a concentration of ± 1·10^6^ cells/mL. After incubation with 10μM INDO-1 AM (Invitrogen) for 30 minutes at 37°C with repeated shaking, the cells were washed and resuspended in HEPES-buffered HBSS with calcium, and kept on ice until use. 100μL of cell suspension was prewarmed in a UV-STAR microtiter black plate (Greiner) at 37°C in the Clariostar Analyser (BMG Labtech, Ortenberg, Germany; excitation 350 nm, emission 395 nm, and 460 nm). In Figure 1, baseline fluorescence (±100–200 s) was measured, cpd3 was added (measured for 150–400 s) and then LPA was added. In **Figure 6**, when necessary, A-96 was added before measurement, baseline fluorescence (±250 sec) was measured, and cpd3 or DMSO was added. Data was calculated by dividing fluorescence emitted at 395 nm (calcium-sensitive) by the fluorescence emitted at 460 nm (calcium-insensitive) for each time point (further described as ratio). The mean baseline ratio was subtracted from the peak effect ratio for each condition (delta ratio).

#### RNA isolation and RT-qPCR

HMC-1, HEK and HEK-LPAR_5_-TRPA1 cells were used to study *LPAR1-6* mRNA expression levels. The day before start of the incubation, ± 5·10^5^ cells per well were seeded in six-well plates. Total RNA was isolated after cell lysis with TRIreagent® (Sigma), chloroform extraction and isopropanol precipitation. After DNase treatment (Promega), cDNA was synthesized by oligo(dT) primers (Invitrogen) and random hexamers (Promega) followed by reverse transcriptase (Fermentas). qPCR analysis was performed using a LightCycler®480 II (Roche) detection system using the SensiFAST™ Sybr No-Rox mix (Bioline). All graphs depict *LPAR1-6* mRNA expression levels normalized to the following reference genes that were proven to be stably expressed throughout experimental conditions: geometric mean of GAPDH and HPRT or 36B4 and HPRT.

Sequences of primers used are *LPAR1*: GGGCCTCATTGACACCAGC (forward) and GGAAAACCGTAATGTGCCTCTC (reverse); *LPAR2*: CCTGGTCAAGACTGTTGTCATC (forward) and GACTCACAGCCTAAACCATCC (reverse); *LPAR3*: GCTGCCGATTTCTTCGCTG (forward) and AGCAGTCAAGCTACTGTCCAG (reverse); *LPAR4*: TCCTTACCAACATCTATGGGAGC (forward) and ACGTTTGGAGAAGCCTTCAAAG (reverse); *LPAR5*: CACTTGGTGGTCTACAGCTTG (forward) and GCGTAGTAGGAGAGACGAACG (reverse); *LPAR6*: GGACAATGTACCCAATCACTCTC (forward) and ACTTCTCCTGACAGACCAGTTT (reverse); *36B4*: TCATCAACGGTACAAACGA (forward) and GCCTTGACCTTTTCAGCAAG (reverse); *GAPDH*: CAAGATCATCAGCAATGCCT (forward) and CAGGGATGATGTTCTGGAGAG (reverse); *HPRT*: TGACCTTGATTTATTTTGCATACC (forward) and CGAGCAAGACGTTCAGTCCT (reverse).

### *In vivo* experiments

#### Experimental animals

Experiments were performed with female C57Bl/6 wild type mice (Janvier), ${\text{sPLA}}_{2}^{\text{tg}}$ mice (23), *Trpa1 KO* mice [Jackson; (24)] and male Swiss wild type mice (Federal University of Parana colony). The ${\text{sPLA}}_{2}^{\text{tg}}$ mouse model contains a transgene for secreted phospholipase A2. The background strain is C57Bl/6. The *Trpa1 KO* mouse background strain is a mix of C57Bl/6J with FVB 129P2/OlaHsd. All mice were aged 8 weeks to 8 months. The difference in age is considered to have no influence on the nociception and itch responses. Mice were housed conventionally with ad libitum water and food, consisting of regular chow (Teklad 2916, Envigo). Experiments were conducted according to the institutional Animal Care and Use Committee regulations. All methods were carried out in accordance with relevant guidelines and regulations. The study was also carried out in compliance with the ARRIVE guidelines.

#### Scratch activity assay

Scratch activity of the animals (${\text{sPLA}}_{2}^{\text{tg}}$*, Trpa1 KO and WT mice)* was measured using an in-house developed system reported in (25, 26) which allows long-term measurements of scratch activity. Teflon coated 5 x 2 mm magnets (VWR European) were implanted subcutaneously under general anesthesia in both hind paws (below the knee) a week before the experiment. Accompanied by a littermate of the same gender without magnets, mice were permanently placed in a cage surrounded by a magnetic coil. An oscillograph attached to a computer registered the electric currents induced by movements of the implanted magnets. Chronic scratch activity was assessed at night (7 pm−7 am) for the duration as described with each experiment. Custom made software was used to quantify scratch and total movements exactly as described in (26). Scratch activity was expressed as the average total duration (in seconds) of scratch movements per 12 h measurement.

Cpd3 was dissolved in vehicle (15% glycofurol, 5% solutol and 40% PEG400) and administered by oral gavage (100 μl) at 17h. Measurements where started at 19h.

#### Formalin test

Male Swiss mice (25 ± 3g) were used and pre-treated 24 h and/or 2 h before assessment of nociceptive responses by oral gavage of vehicle or cpd3. Mice that received morphine (s.c.) were treated 30 min before the test. The treatments were performed in a blinded manner. After the treatments, the mice were habituated in the acrylic chambers during 10 min, then received formalin (5%/10 μL) or vehicle (saline, 10 μL) injection into the right hindpaw, and were returned immediately to the chambers for behavioral analyses. The time animals spent licking the injected paw was registered for 50 min, which was counted in 5-min intervals. Phase I of the formalin response was considered the interval from 0–5 min, while phase II was the interval from 15 to 30 min.

#### Paw withdrawal test

Male Swiss mice (25 ± 3g) were treated 24 h beforehand and again on the subsequent day, after the baseline measurement with oral gavage of cpd3. The treatments were performed in a blinded manner. Mice were habituated for about 2 h in acrylic chambers and the baseline paw withdrawal threshold (in gram) was assessed by an electronic version of von Frey filaments. A handheld force transducer (electronic anesthesiometer; Insight, Ribeirão Preto, SP, Brazil) adapted with a 0.5 mm^2^ polypropylene tip was used to evoke hind paw nociceptive withdrawal response. The intensity of the pressure (in gram) at the moment of paw withdrawal was automatically recorded. Carrageenan, PGE_2_, or saline was injected into the right hindpaw and the paw withdrawal threshold was assessed hourly up to 6 hours.

### Statistical analyses

GraphPad Prism (Version 8.3.0 for Mac OS X, GraphPad Software, La Jolla, California USA) was used for statistical analyses. For nociceptive behavior, time course results were analyzed by two-way ANOVA followed by Bonferroni *post hoc* test. Cumulative time results were analyzed by one-way ANOVA followed by Bonferroni *post hoc* test. Both were expressed as the F-ratio with degrees of freedom for the numerator and denominator, followed by the *p*-value. For analysis of chronic scratch activity, differences between two groups under the same condition were tested by unpaired *t*-test, differences within the same group under different consecutive conditions by paired *t*-test. Results were considered statistically significant when *p* < 0.05.

## Results

### Cpd3 inhibits LPAR_5_ in HMC-1 cells

Cpd3 has been developed as an LPAR_5_-antagonist by Kozian and colleagues (20). To verify this function of cpd3, we cultured HMC-1 cells, which express high levels of *LPAR*_5_ (Figure 1B) (27), and triggered a calcium response with the agonist LPA 18:1 (1 μM). Addition of LPA in the absence of cpd3, led to an induction of free intracellular calcium. As expected, the release of calcium was diminished with increasing amounts of the LPAR_5_-antagonist cpd3 (Figure 1A; Supplementary Figure 1).

### Cpd3 reduces inflammatory nociceptive behavior induced by formalin

We tested the effects of the LPAR_5_ inhibitor cpd3 on formalin-induced nociception. Male Swiss mice were treated with a single dose of vehicle, cpd3 (11 mg/kg p.o., 2 h before measurement), or morphine (5 mg/kg, s.c., 30 min before measurement). At starting point, the mice received formalin (5%/10 μL) or vehicle (saline, 10 μL) injection into the right hindpaw. The amount of licking time was measured for 50 min.

Injection of formalin into the hindpaw evoked an increase in licking behavior compared to saline-injected mice. A single dose of cpd3 (11 mg/kg) did not reduce licking behavior, but did induce a small delay in the onset of the formalin effect (Supplementary Figure 2). In order to reach a higher concentration of cpd3 in the circulation, we subsequently treated male Swiss mice twice with vehicle or cpd3 (11 mg/kg, p.o., i.e. 24 h and 2 h before formalin injection). Mice received formalin (5%/10 μL) or vehicle (saline, 10 μL) injection into the right hindpaw.

**Figure 2:**
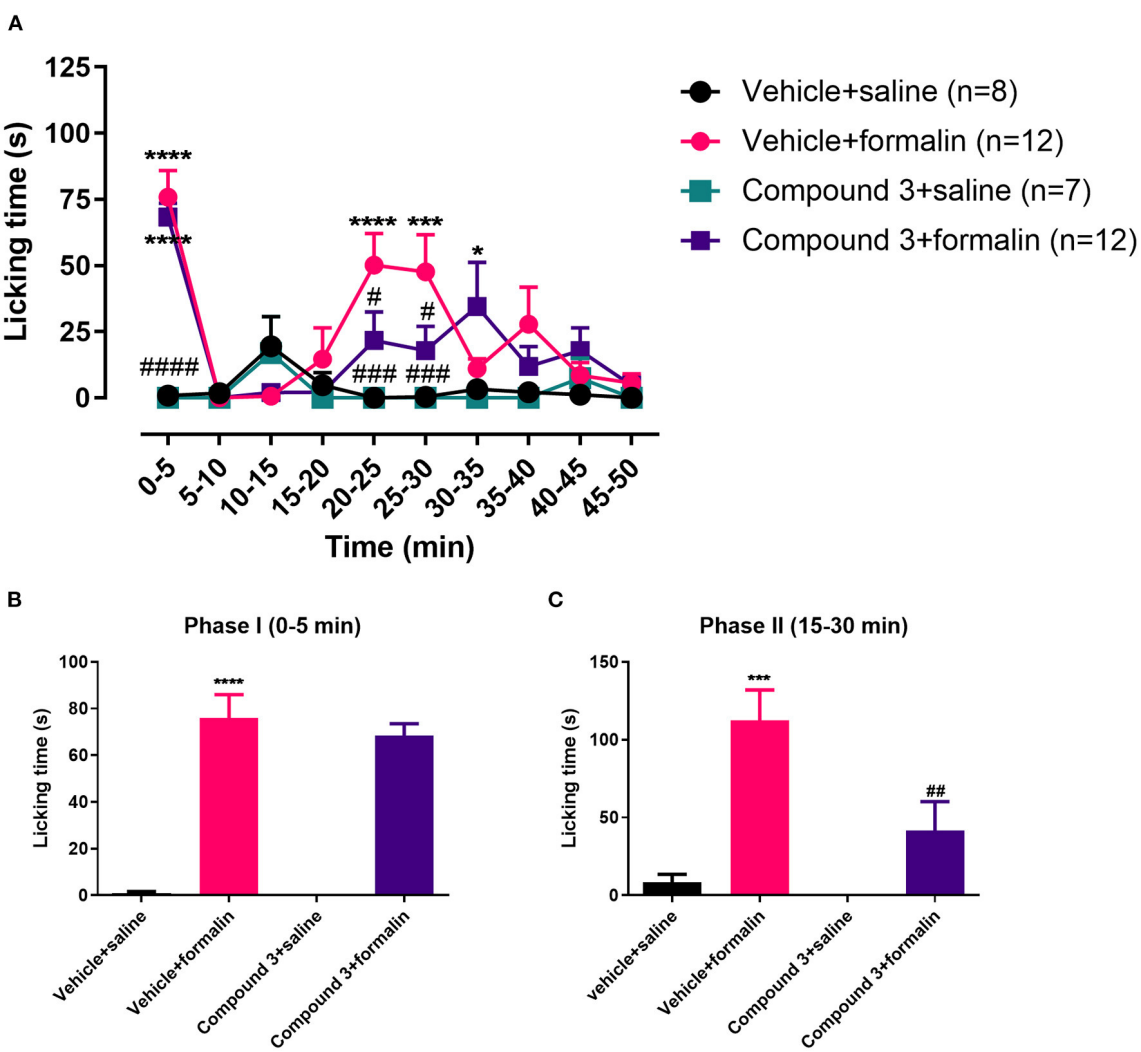
Time course **(A)** and cumulative licking time **(B,C)** induced by formalin injected in the hindpaw, after pre-treatment with a double dose of vehicle or cpd3 (11 mg/kg). **(A)** Two-way ANOVA followed by Bonferroni *post hoc* test. **(B,C)** One-way ANOVA followed by Bonferroni *post hoc* test. *p < 0.05; ***p < 0.001; ****p < 0.0001 compared to vehicle + saline group. ^#^*p* < 0.05; ^###^*p* < 0.001; ^####^*p* < 0.0001 compared to vehicle + formalin group.

Injection of formalin into the hindpaw evoked an increase in licking behavior in both phase I [F (3, 34) = 30.68; *p* < 0.0001] and phase II [F (3, 34) = 8.945; *p* = 0.0004], which was significantly different from control mice (vehicle + saline; Figure 2). Pre-treatment with cpd3 did not change the licking response evoked by formalin in phase I [F (3, 34) = 32.99; *p* > 0.9999]. However, in phase II, pre-treatment with a double dose of cpd3 did reduce the licking response compared to vehicle treated mice [F (3, 34) = 8.945; p = 0.0079] (Figures 2B,C). These data indicate that cpd3 has anti-nociceptive effects affecting phase II, which may be related to modulation of inflammatory pain. Similar to pre-treatment with a single dose of cpd3, a double dose of cpd3 caused a delay in the onset of the formalin effect (Figure 2A).

### High single dosage of cpd3 reduces inflammatory nociceptive behavior in both phase I and II

**Figure 3:**
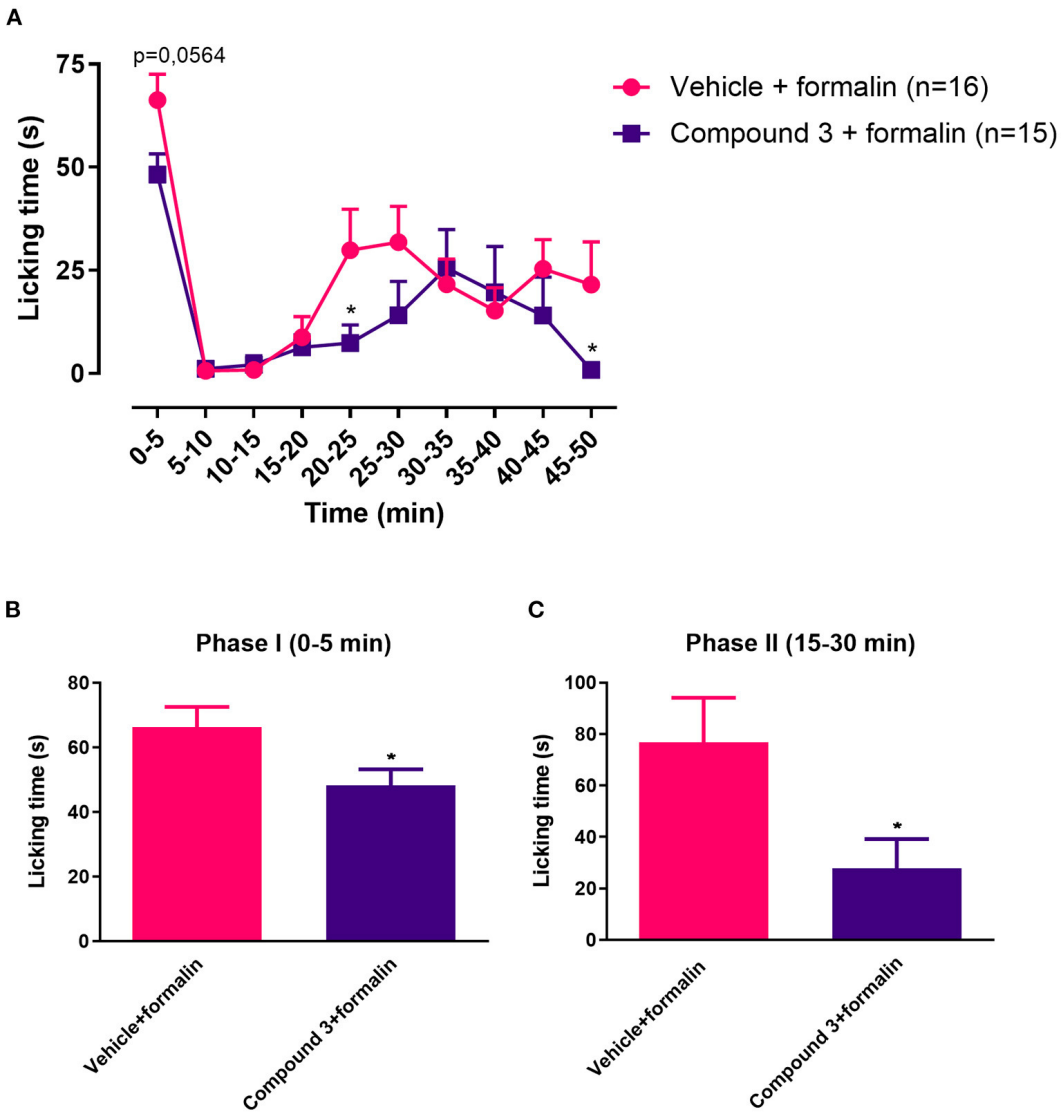
Time course **(A)** and cumulative licking time **(B,C)** induced by formalin injected in the hindpaw, after pre-treatment with a single dose of vehicle or cpd3 (110 mg/kg). **(A)** Multiple unpaired *T*-test of Two-way ANOVA. **(B,C)** *T*-test, *p < 0.05 compared to vehicle + formalin group.

In the results above, a single dose of 11 mg/kg cpd3 had no effect on licking time induced by formalin injection, whereas a double dose of 11 mg/kg cpd3 reduced the licking time in phase II. Therefore, we wanted to measure the effect of a single, higher dose of cpd3 (110 mg/kg, p.o.), 2 h before measurement. Increased licking behavior in phase I and phase II, induced by injection of formalin, was significantly reduced in both phases by a single high dose of cpd3 compared to vehicle-treated rats [phase I, t (29) = 2.238; *p* = 0.0331; phase II, t (29) = 2.342; p = 0.0263] (Figure 3).

### Cpd3 reduces hyperalgesia induced by carrageenan or PGE_2_

Besides formalin-induced inflammatory nociception, we also studied the effect of cpd3 on inflammatory mechanical hyperalgesia. Activation of LPAR_5_ results in an intracellular increase of cAMP (8), which increases CREB phosphorylation and thereby induces hyperalgesia *via* central sensitization (15, 28, 29). Intraplantar injection of carrageenan induces mechanical hyperalgesia in mice by triggering a cytokine cascade initiated by TNF-α and CXCL1 production, which stimulate the release of IL−1β and subsequent PGE_2_ production (30). In turn, PGE_2_ sensitizes the nociceptor, by increasing intracellular cAMP content, which can be detected as mechanical hyperalgesia (31, 32).

**Figure 4:**
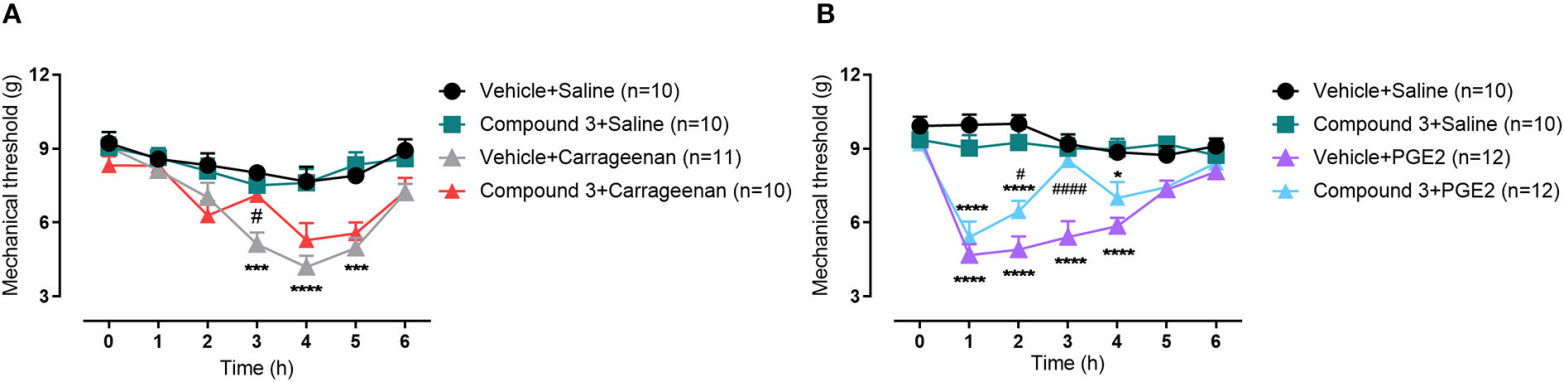
Time course of paw withdrawal threshold (g) induced by **(A)** carrageenan (200 μg/20μL) or **(B)** PGE_2_ (1 nmol/10 μL) injected in the hindpaw, after pre-treatment with a double dose of vehicle or cpd3 (11 mg/kg, p.o.). *p < 0.05; ***p < 0.001; ****p < 0.0001 compared to vehicle + saline group. ^#^*p* < 0.05; ^####^*p* < 0.0001 compared to vehicle + carrageenan or vehicle + PGE_2_ group. Two-way ANOVA followed by Bonferroni post hoc test.

In this experiment, inhibition of LPAR_5_ by cpd3 was analyzed during hyperalgesia induced with carrageenan or PGE_2_ after which the paw withdrawal threshold was measured. Male Swiss mice were treated twice with vehicle or cpd3 (11 mg/kg, p.o.) 24 h before as well as right before measuring (after baseline measurement). Carrageenan (200 μg/20μL), PGE_2_ (1 nmol/10 μL) or saline (10 μL) was injected into the right hindpaw and the paw withdrawal threshold was assessed hourly up to 6 hours. Both carrageenan and PGE_2_ treatment led to a strongly decreased paw withdrawal threshold compared to saline, indicating mechanical hyperalgesia, which was diminished by pre-treatment with cpd3 [Figure 4A; F (18, 222) = 2,548; *p* = 0.0008], [Figure 4B; F (18, 240) = 7,636; *p* < 0.0001]. Cpd3 pre-treatment with subsequent saline injection had no effect on the paw withdrawal threshold. These results indicate that cpd3 diminishes hyperalgesia induced by carrageenan or PGE_2_.

### Cpd3 increases scratch activity in *${\mathrm{sPLA}}_{2}^{tg}$* and WT mice

LPAR_5_ is not only involved in pain but also in itch perception (16). We hypothesized that LPAR_5_-inhibition by cpd3 would reduce scratch activity in mice. In previous studies on itch in mouse models, we have characterized phospholipase A2 transgenic mice (${\text{sPLA}}_{2}^{\text{tg}}$) as a mouse model of chronic itch, which overexpress secreted PLA_2_ in, amongst other tissues, the skin. These mice have a skin phenotype due to this high sPLA_2_ activity and subsequent increased production of LPA could contribute to this phenotype (23). Hypothetically, high levels of LPA produced by sPLA_2_ could bind to LPAR_5_ and thereby induce itch signaling. Hence, inhibition of LPAR_5_ by cpd3 should reduce the scratch activity in ${\text{sPLA}}_{2}^{\text{tg}}$ mice to a level similar to WT mice.

**Figure 5:**
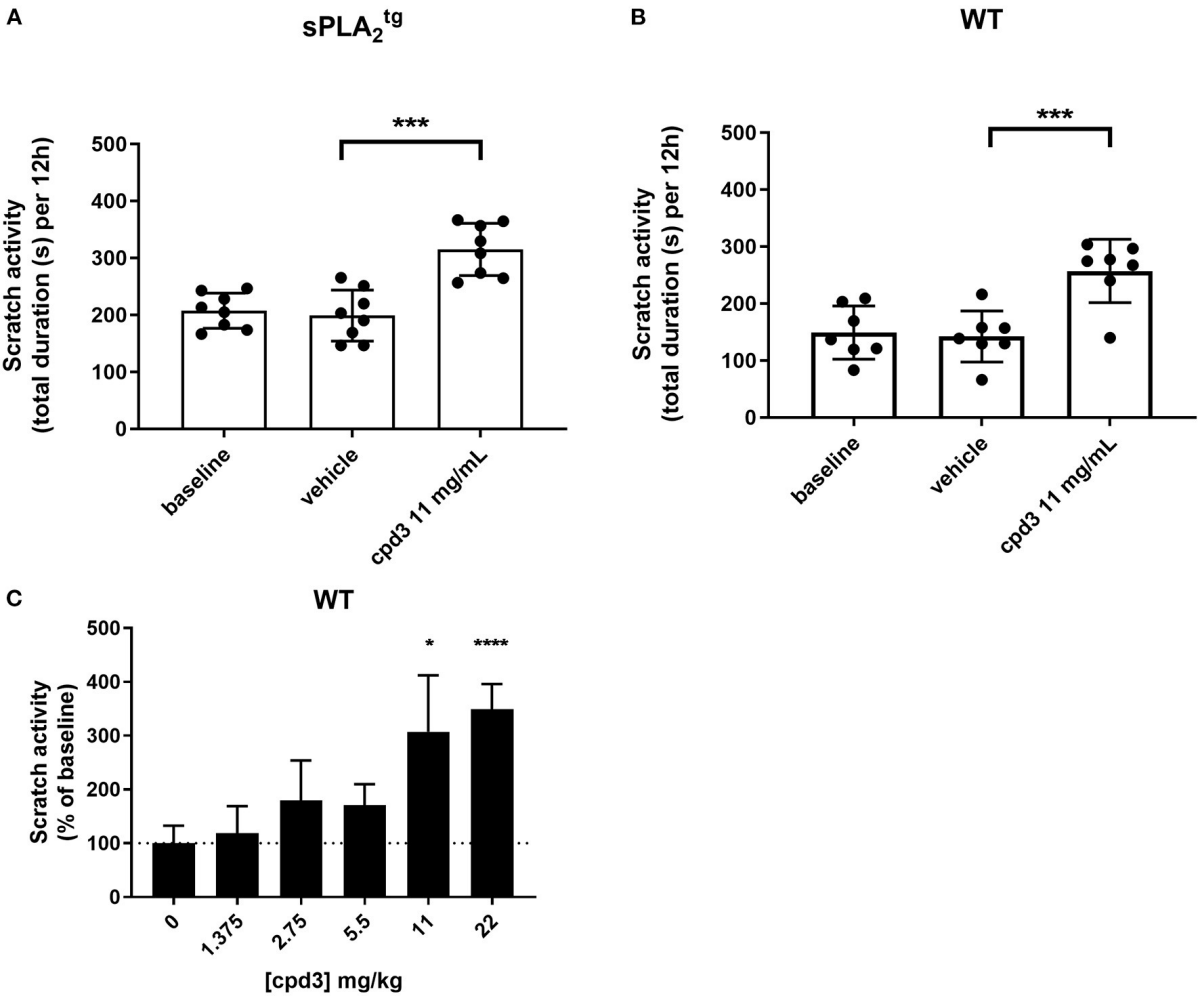
Scratch activity (total duration in seconds per 12 h, mean of four consecutive nights) in **(A)** ${\mathrm{sPLA}}_{2}^{tg}$ mice (*n* = 8), **(B)** wild type (*n* = 7), receiving oral gavage of vehicle and subsequently cpd3 (11 mg/kg) and **(C)** wild type (*n* = 4 per group), during 12 hours after a single oral gavage of different doses of cpd3. Statistics: **(A,B)** paired *t*-test; ***p < 0.001, **(C)** paired t-test compared to baseline; *p < 0.05; ****p < 0.0001.

In both ${\text{sPLA}}_{2}^{\text{tg}}$ and WT mice, basal scratch activity during four consecutive nights was measured without any intervention. The baseline scratch activity (mean of four nights) was significantly higher in ${\text{sPLA}}_{2}^{\text{tg}}$ mice (200 s Figure 5A baseline), compared to WT mice (150 s Figure 5B baseline) (unpaired *t*-test, *p* = 0.0125). This baseline difference might be due to the overexpression of secreted PLA_2_ in these mice, resulting in epidermal and adnexal hyperplasia, hyperkeratosis and almost total alopecia (23). Strikingly, in both ${\mathrm{sPLA}}_{2}^{tg}$ and WT mice, oral administration of 11 mg/kg cpd3 showed a strong increase in scratch activity, instead of the expected decrease (Figures 5A,B). The mice showed a strong increase in scratch activity in the first night, which diminished during the following nights but remained higher than before administration (Supplementary Figure 3). We investigated the dose-dependent cpd3-induced scratch activity by treating five groups of naïve WT mice with a single dose of cpd3, containing increasing concentrations. Figure 5C shows that increasing concentrations of cpd3 from 1.375 up to 22 mg/kg, induced scratch activity up to 350% of baseline. These results suggest that cpd3 is a strong pruritogen rather than an antagonist of itch receptors.

### Cpd3 activates TRPA1

**Figure 6:**
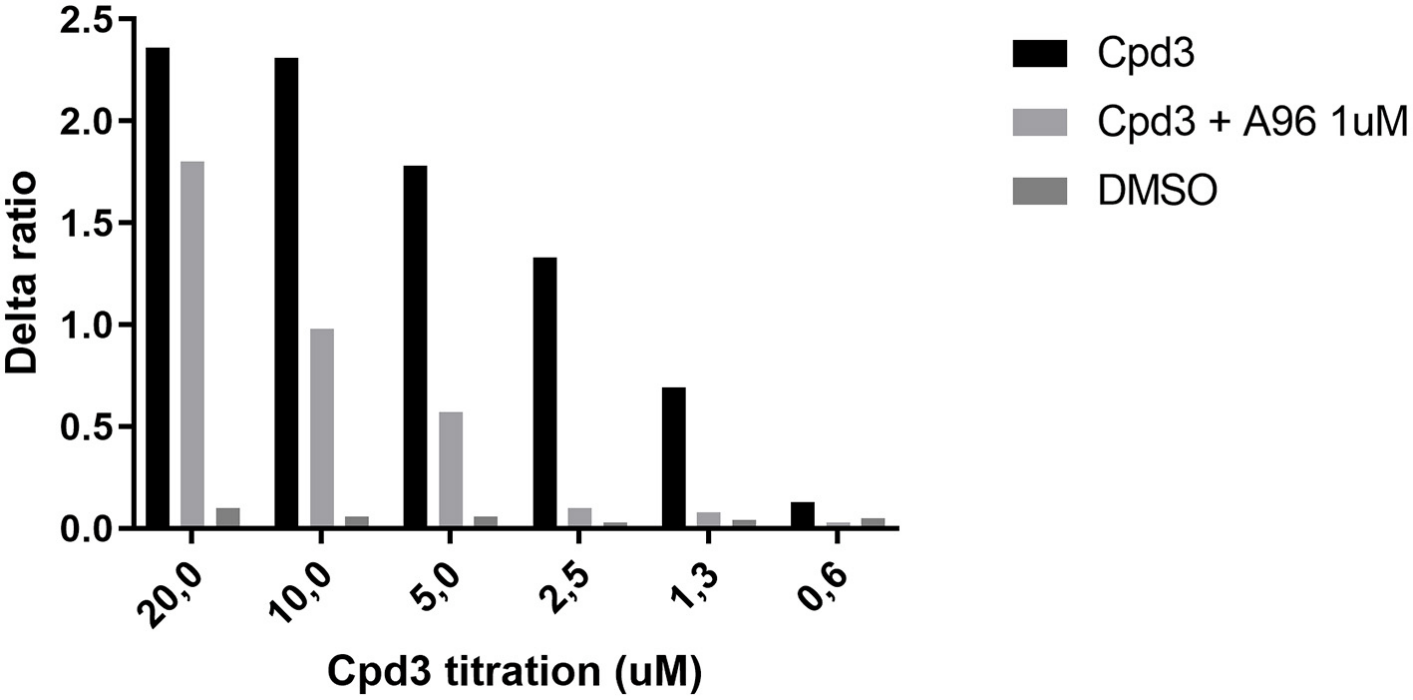
Increase of free Ca^2+^ in HEK-TRPA1 cells, measuring the effect of different concentrations of Cpd3, Cpd3 + TRPA1-antagonist A-967079, and DMSO control (max. final concentration 0.2%). Delta ratio indicates the ratio of emitted Indo-1 fluorescence (395/460 nm).

Based on the *in vivo* experiments, cpd3 appears to be an inducer of itch. One of the most prominent itch channels is the transient receptor potential ankyrin 1 (TRPA1) channel, located on sensory nerve endings (33, 34). Here, we used HEK cells with inducible TRPA1 expression and measured the free intracellular calcium induced by cpd3 administration, in the presence and absence of the TRPA1-antagonist A-967079 (A-96). Figure 6 shows a strong calcium releasing effect of cpd3 on HEK-TRPA1 cells. The competitive TRPA1-antagonist A-967079 (1 μM) was able to inhibit the effects of cpd3 completely at concentrations below 2.5 μM. The vehicle DMSO did not affect intracellular free calcium. Cpd3 also did not give a calcium response in non-transfected cells nor in cells transfected with TRPV1 (not shown).

### Cpd3 also induces scratch activity in *Trpa1 KO* mice

**Figure 7:**
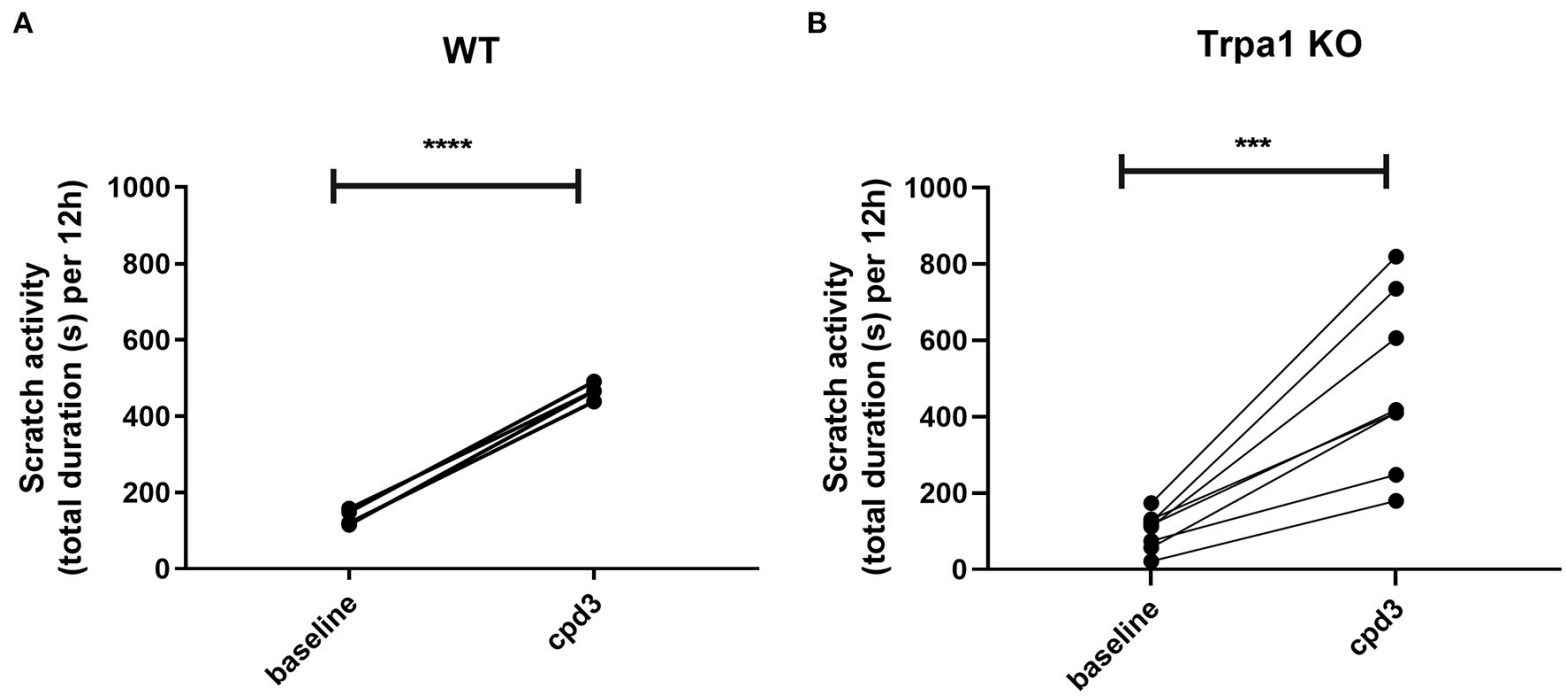
Scratch activity (total duration in seconds per 12 h) in **(A)** wild type (*n* = 4) and **(B)** *Trpa1 KO* mice (*n* = 7) receiving a single dose of 22 mg/kg cpd3 by oral gavage. Statistics: paired *t*-test; ***p < 0.001; ****p < 0.0001.

To establish the itch-signaling pathway of cpd3 on TRPA1 *in vivo*, we orally administered cpd3 at a concentration of 22 mg/kg to WT mice and to Trpa1 knockout mice (*Trpa1 KO*) and measured the duration of scratch activity for 12 h during the night. Figure 7A shows the increase of scratch activity in WT mice after oral gavage of 22 mg/kg cpd3. Surprisingly, in *Trpa1 KO* mice, cpd3 also induced a significant increase in scratch activity (Figure 7B). This suggests that the scratch-inducing effects of cpd3 are conducted by more factors than just TRPA1.

## Discussion

Cpd3 has been developed as an LPAR_5_-antagonist to inhibit LPA signaling in mast cells and microglia, which play a critical role in innate immunity and inflammation (20). In inflammatory research, cpd3 can prevent LPAR_5_-mediated suppression of signaling in mouse CD8 T cells (35). Recently, it was also shown that cpd3 could be a versatile pharmacological tool to regulate inflammatory signaling in BV-2 microglia cells (36).

Next to inflammation, LPAR_5_-signaling also plays a prominent role in sensory activation (17, 37, 38). In the study of Lin et al., *Lpar5*-deficient mice were protected from neuropathic pain induced by partial sciatic nerve ligation (15). In a second, independent study, these mice also showed decreased pain sensitivity in tail withdrawal tests and faster recovery after inflammatory pain induced by complete Freund's adjuvant (14). *Lpar5* gene expression is found in dorsal root ganglion neurons. It is both a G_α12/13_- and G_q_-coupled receptor, thereby able to increase cAMP production (8, 15).

In our previous work, we have shown that LPA concentrations were increased in the sera of women with intrahepatic cholestasis of pregnancy (ICP, which is characterized by chronic pruritus), and intradermal injection of LPA in mice induced an increased scratch response (25). Kittaka et al. (16) showed that of all six LPA receptors, LPAR_5_ is the most prominent receptor involved in itch signaling. LPAR_5_ stimulation causes intracellular activation of phospholipase A2 and phospholipase D, leading to intracellular LPA release, activating TRPA1 and TRPV1 channels and thereby eliciting itch sensation. LPA-induced itch-related behaviors are decreased in TRPA1 and TRPV1 deficient mice (16).

So far, cpd3 has not been studied in terms of sensory signaling. Therefore, we tested the effect of cpd3 on established pain models. The formalin test has been used for chemical pain assessment for many years (39). Formalin produces a distinct biphasic excitatory response in dorsal horn neurons. It shows an immediate acute or phasic peak of neuronal firing 0–10 min post injection, followed by a quiescent period (5 min) and a more prolonged tonic excitatory response over a period of 20–60 min after formalin injection (40). The acute phase is suggested to be due to direct activation of peripheral nociceptors and is susceptible to inhibition by local anesthetic agents and opioids. The late phase would be a response to inflammatory pain that can be inhibited by anti-inflammatory drugs and opioids (41).

In this study, we show that inflammatory nociception, induced by formalin injection, can be reduced by cpd3, when administered twice daily, or with a single high dose (Figures 2, 3). This finding corroborates previous evidence that LPAR_5_-antagonists may reduce inflammatory pain (17). In the presence of cpd3, a delay can be noted in the onset of the formalin effect. This might be due to the anti-inflammatory effect of cpd3 which reduces peripheral sensitization and consequently, delays the start of central sensitization. Secondly, we show that cpd3 reduces mechanical hyperalgesia, induced by carrageenan or PGE_2_ (Figure 4). Hyperalgesia is described as an abnormally increased sensitivity to pain, which may be caused by sensitization of nociceptors or damage to peripheral nerves and can cause hypersensitivity to a stimulus (42–44). Intraplantar injection of carrageenan is extensively used as a model of inflammatory hyperalgesia. It induces mechanical hyperalgesia in mice by triggering a cytokine cascade that culminates in stimulation of COX-2 expression and PGE_2_ production (30, 45). Cpd3 administration restored the paw withdrawal threshold in carrageenan treated-mice, indicating a possible effect in this pathway. In addition, cpd3 caused a significant and sustained reversal of PGE_2_-induced hyperalgesia, suggesting that it causes analgesia by interfering with the hyperalgesic action of the final mediator (i.e. PGE_2_), but not in an upstream step of the cascade. However, it has been reported that some LPAR_5_-antagonists are capable to reduce the expression of pro-inflammatory markers, including COX-2 (36). Peripherally, one mechanism of PGE_2_ induced hyperalgesia is activation of EP_2_ and EP_4_ receptors, which are coupled to G_S_ protein and, when activated, stimulate the cAMP/PKA pathway (32). Moreover, intrathecal PGE_2_ causes hyperalgesia by facilitating glutamate release from presynaptic terminals in the spinal dorsal horn via EP_1_ receptors (46, 47). In the study of Murai et al., it has been shown that the LPAR_5_-antagonist AS2717638 significantly reduced mechanical allodynia induced by intrathecal PGE_2_ in mice (17). Altogether, these results indicate that LPAR_5_-antagonists may interfere with PGE_2_ induced hyperalgesia in the periphery as well as in the spinal cord. Our results are consistent with the study of Murai et al., in which AS2717638 could ameliorate both static mechanical allodynia and thermal hyperalgesia in a rat model of chronic constriction injury (CCI)-induced neuropathic pain (17). Together, they show the efficacy of LPAR_5_-antagonists in the treatment against inflammatory and neuropathic pain.

Notably, in this study we also show that cpd3 induces scratch activity in mice. We hypothesized that cpd3 would inhibit scratch activity similar to pain perception. In a mouse model for chronic itch (${\text{sPLA}}_{2}^{\text{tg}}$ mice), administration of cpd3 was expected to reduce scratch activity to the level of WT mice. However, cpd3 administration showed a surprising difference in itch perception compared to pain perception. Scratch activity was increased rather than reduced in both ${\text{sPLA}}_{2}^{\text{tg}}$ and WT mice after administration of cpd3. This increase happened in a dose-dependent manner (Figure 5). The results also showed that the induction of scratch activity by cpd3 is in part a transient phenomenon (Supplementary Figure 3). Administration of cpd3 caused the strongest increase in scratch activity at the first day, after which the activity decreased on subsequent days with administration of the same concentration. This suggests that involved receptors may get desensitized. However, this desensitization is only partly, since the scratch activity flattens at a level that is still higher compared to baseline, indicating a sustained increase in scratch activity after cpd3 administration. Together, these results suggest that, independent of the inhibition on LPAR_5_, cpd3 has a second, scratch inducing way of action.

One of the sensory channels involved in itch signaling is TRPA1. Opening of this channel leads to an influx of cations, including calcium and when located on nerve endings, this might contribute to eliciting an action potential, leading to itch sensation. Cpd3 appears to be a very strong TRPA1 agonist (Figure 6). Therefore, we administered cpd3 to *Trpa1 KO* mice, and hypothesized that the scratch activity would remain unchanged. However, Figure 7 shows that cpd3 administration induces scratch activity in *Trpa1 KO* mice as well. This suggests that TRPA1 is not the only sensory channel that can be activated by cpd3 and induce scratch activity. The pruriceptor TRPV1 is not likely to be involved since we did not see *in vitro* activation of TRPV1-expressing cells upon cpd3 addition. Other pruriceptors such as those from the MRGPR family could be involved.

This study showed that LPAR_5_ antagonism can reduce nociception. Hence, LPAR_5_-antagonists like cpd3 could be mediators in the reduction of inflammatory pain and hyperalgesia, but more selective compounds need to be designed to avoid off-target pruritogenic effects.

## Data availability statement

The raw data supporting the conclusions of this article will be made available by the authors, without undue reservation.

## Ethics statement

The animal study was reviewed and approved by Institutional Animal Care and Use Committee of the University of Amsterdam (Amsterdam, Netherlands) and the Federal University of Parana (Brasil) Ethics Committee.

## Author contributions

JL wrote the manuscript with intellectual guidance by MN, HB, JS, JC, and RO. Experiments were designed by JL, JC, and RO. Experiments were performed and data was analyzed by JL, EA, AB, DT, JC, and RO. All authors contributed to the article and approved the submitted version.

## Funding

This research was funded by a TOP ZonMW Grant (#40-00812-98-10054) and a grant from the Dioraphte Foundation (#1115) to RO.

## Conflict of interest

The authors declare that the research was conducted in the absence of any commercial or financial relationships that could be construed as a potential conflict of interest.

## Publisher's note

All claims expressed in this article are solely those of the authors and do not necessarily represent those of their affiliated organizations, or those of the publisher, the editors and the reviewers. Any product that may be evaluated in this article, or claim that may be made by its manufacturer, is not guaranteed or endorsed by the publisher.

## Abbreviations

BV-2 cell: microglia cell
cAMP: cyclic adenosine monophosphate
Cpd3: compound 3
CREB: cAMP response element-binding protein
DMSO: dimethyl sulfoxide
HEK cell: human embryonic kidney cell
HMC-1: human mast cell
LPA: lysophosphatidic acid
LPAR_5_: lysophosphatidic acid receptor 5
PGE_2_: prostaglandin E2
PKA: protein kinase A
PKC: protein kinase C
sPLA_2_: secreted phospholipase A2
TRPA1: transient receptor potential ankyrin 1
TRPV1: transient receptor potential vanilloid 1.
