# Supplementary material for: An LPAR5-antagonist that reduces nociception and increases pruriception
Source: Front Pain Res (Lausanne). 2022 Jul 26;3:963174. doi: 10.3389/fpain.2022.963174 (PMC9360597; doi:10.3389/fpain.2022.963174)
Supplement: Supplementary file 1 [file Data_Sheet_1.pdf]

Supplementary Figures

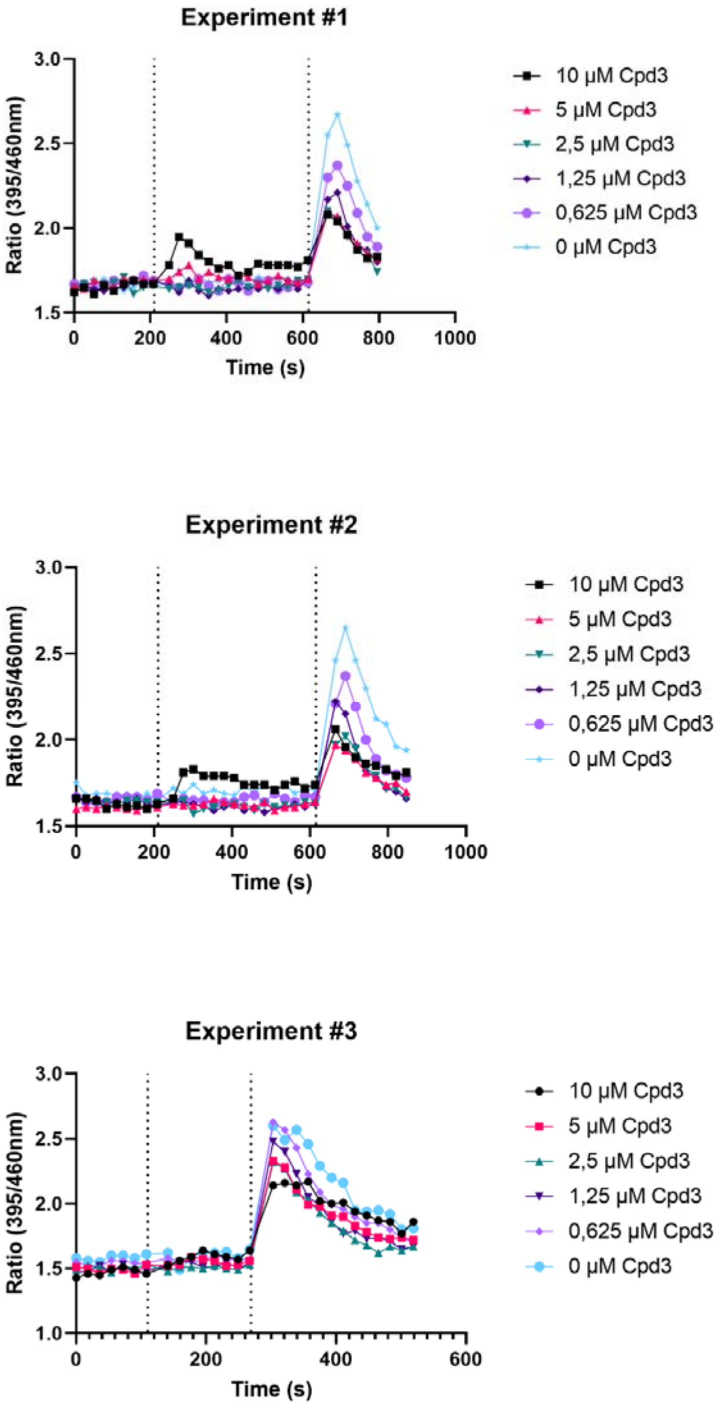

Supplementary Figure 1: Ca^{2+} release in HMC-1 cells over time, indicated by the ratio of 395nm (calcium-sensitive) over 460nm (calcium-insensitive). Measuring baseline (±100-200 sec), addition of different concentrations of cpd3 (measured for 150-400 sec) and addition of 1µM LPA 18:1.

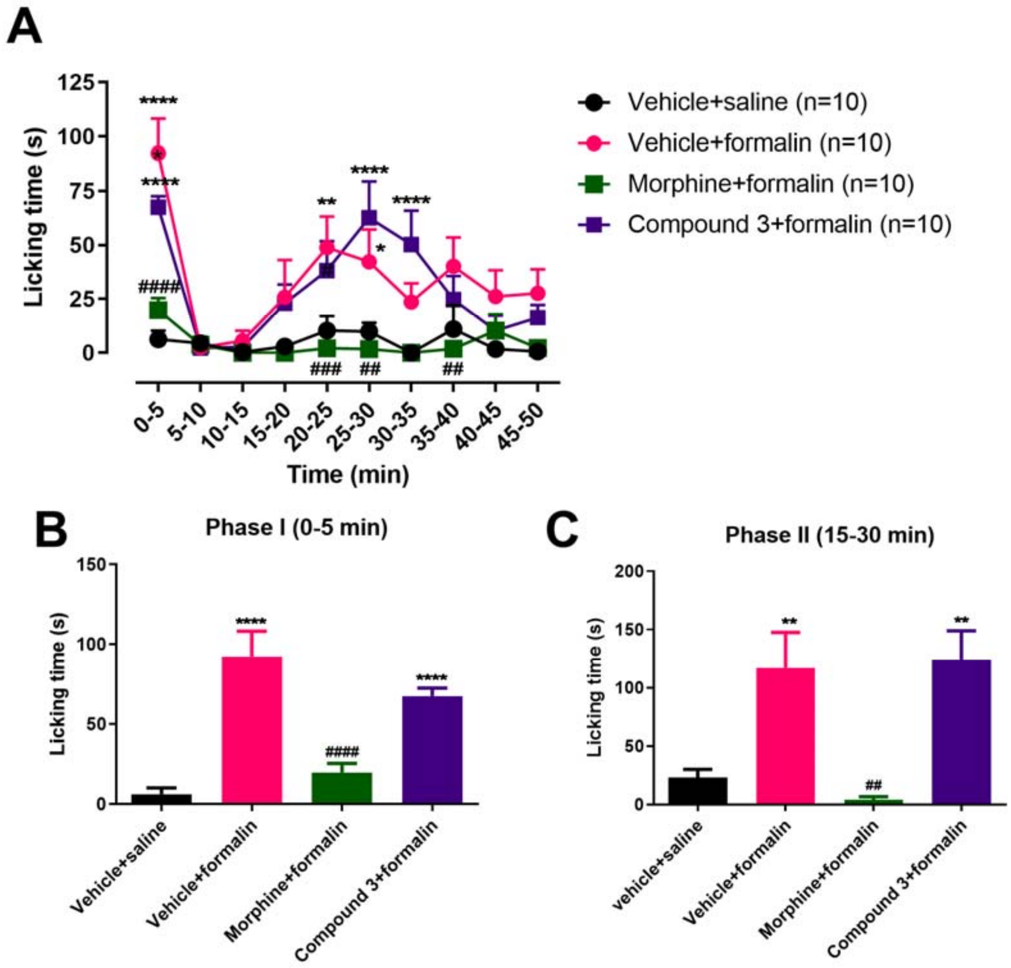

Supplementary Figure 2: Single and low dose administration of cpd3 does not significantly affect formalin-induced licking behavior.

Time course (A) and cumulative licking time (B,C) induced by formalin injected in the hindpaw, after pre-treatment with a single dose of vehicle, morphine (5 mg/kg s.c.) or cpd3 (11 mg/kg p.o.). (A) Two-way ANOVA followed by Bonferroni post hoc test. (B,C) One-way ANOVA followed by Bonferroni post hoc test. * $p<0.05$; ** $P<0.01$; **** $p<0.0001$ compared to vehicle + saline group. ## $p<0.01$; ### $p<0.001$; #### $p<0.0001$ compared to vehicle + formalin group.

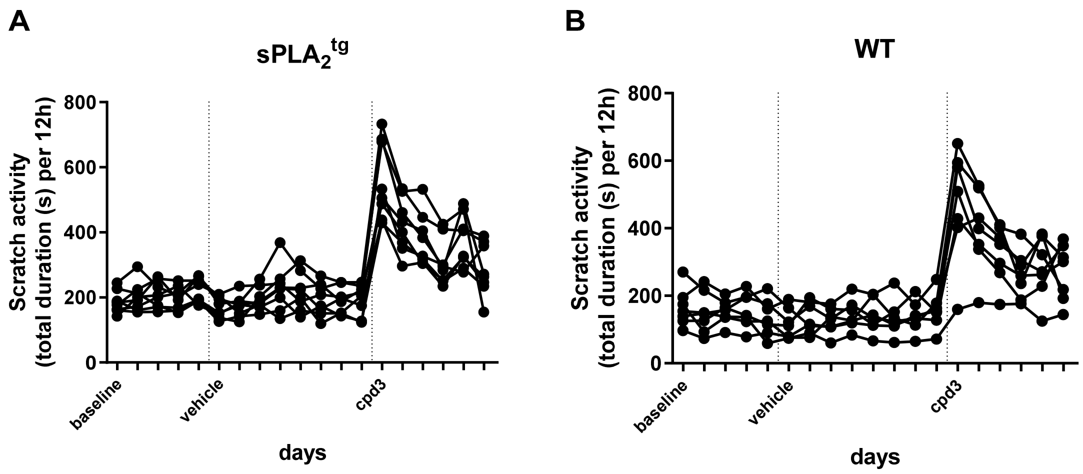

Supplementary Figure 3: Scratch activity in *sPLA₂ᵗᵍ* mice (n=8) (**A**) and wild type (n=7) (**B**), receiving oral gavage of vehicle and cpd3 (11 mg/kg). Same experiment as in Figure 5.
